# Enhanced photocatalytic hydrogen evolution by combining water soluble graphene with cobalt salts

**DOI:** 10.3762/bjnano.5.128

**Published:** 2014-07-29

**Authors:** Jing Wang, Ke Feng, Hui-Hui Zhang, Bin Chen, Zhi-Jun Li, Qing-Yuan Meng, Li-Ping Zhang, Chen-Ho Tung, Li-Zhu Wu

**Affiliations:** 1 Key Laboratory of Photochemical Conversion and Optoelectronic Materials, Technical Institute of Physics and Chemistry & University of Chinese Academy of Sciences, the Chinese Academy of Sciences, Beijing 100190, P. R. China

**Keywords:** cobalt salts, earth-abundant catalyst, photocatalysis, photocatalytic hydrogen evolution, water-dispersible sulfonated-graphene

## Abstract

There is tremendous effort put in the pursuit for cheap and efficient catalysts for photocatalytic hydrogen evolution systems. Herein, we report an active catalyst that uses the earth-abundant element cobalt and water-dispersible sulfonated graphene. The photocatalytic hydrogen evolution activity of the catalyst was tested by using triethanolamine (TEOA) as electron donor and eosin Y (EY) as the photosensitizer under LED irradiation at 525 nm. Hydrogen was produced constantly even after 20 h, and the turnover number (TON) reached 148 (H_2_/Co) in 4 h with respect to the initial concentration of the added cobalt salts was shown to be 5.6 times larger than that without graphene.

## Introduction

Photocatalytic hydrogen evolution from water-splitting is a long-standing goal for researchers since it can help to supply the growing worldwide energy demand not only environmentally friendly but also sustainably [1–4]. Platinum, the most efficient hydrogen evolution co-catalyst, is rare and expensive, which limits its availability [5]. Hence, developing photocatalytic systems that rely only on earth-abundant elements are desired for making hydrogen a competitive alternative energy source. In recent years, systems based on iron complexes, nickel complexes or molybdenum complexes have been reported as promising candidates for catalyzing the hydrogen evolution [6–15]. Cobalt-based catalysts are particularly attractive catalysts that are easily obtained, environmentally benign and rely on earth-abundant elements [16]. Molecular cobalt catalysts [17], such as polypyridyl complexes [18–19], oxime complexes [20], have been proven to be efficient in the photocatalytic production of hydrogen, and the turnover number (TON) has become higher upon introducing more appropriate ligands. Besides, cobalt-based heterogeneous structures are also of interest [21–22]. A hybrid Co_h_–CdTe artificial catalyst for photocatalytic hydrogen evolution [23], for example, was simply constructed in situ from earth-abundant cobalt salts and CdTe quantum dots.

As a new carbon material with large surface area and excellent electrical properties, graphene has raised much attention since 2004 [24–33]. Specifically, graphene has been involved in photocatalytic hydrogen production systems [34], such as TiO_2_-(N)RGO-Pt [35–38], g-C_3_N_4_-RGO-Pt [39], CdS-RGO-Pt [40–43], MoS_2_-NRGO [44–45], EY-RGO-Pt [46] and BiVO_4_-RGO-Ru/SrTiO_3_:Rh [47] (RGO: reduced graphene oxide; EY: eosin Y). Graphene enhances the catalytic efficiency of hydrogen evolution remarkably. By using transient photovoltage and photocurrent techniques [48–50], the function of graphene was examined. More recently, our group has demonstrated the efficient forward electron-transfer mediated by graphene in terms of the unique spectroscopic property of photosensitizer EY [51]. The result stimulated us to explore graphene-based hydrogen evolution systems with earth-abundant co-catalysts.

In the present work, we report a new water-soluble graphene–cobalt-based hydrogen evolution system, showing a 5.6 times higher efficiency than that of the same system without graphene. Herein, sulfonated-graphene (G-SO_3_), being water-soluble and partially reduced [52–53], serves as a great platform [41,51] to support the catalysts. With TEOA (triethanolamine) as an electron donor, EY as a photosensitizer, Co(TEOA)_2_^2+^ is formed in situ and adsorbed at the surface or around the G-SO_3_ when cobalt salts and G-SO_3_ are introduced into the hydrogen evolution system. Upon irradiation by visible light (525 nm LEDs as light source) for 4 h, the system is able to produce hydrogen with a TON up to 148 with the initial concentration of cobalt salts added. And hydrogen constantly evolves even after 20 h irradiation.

## Results and Discussion

**Figure 1:**
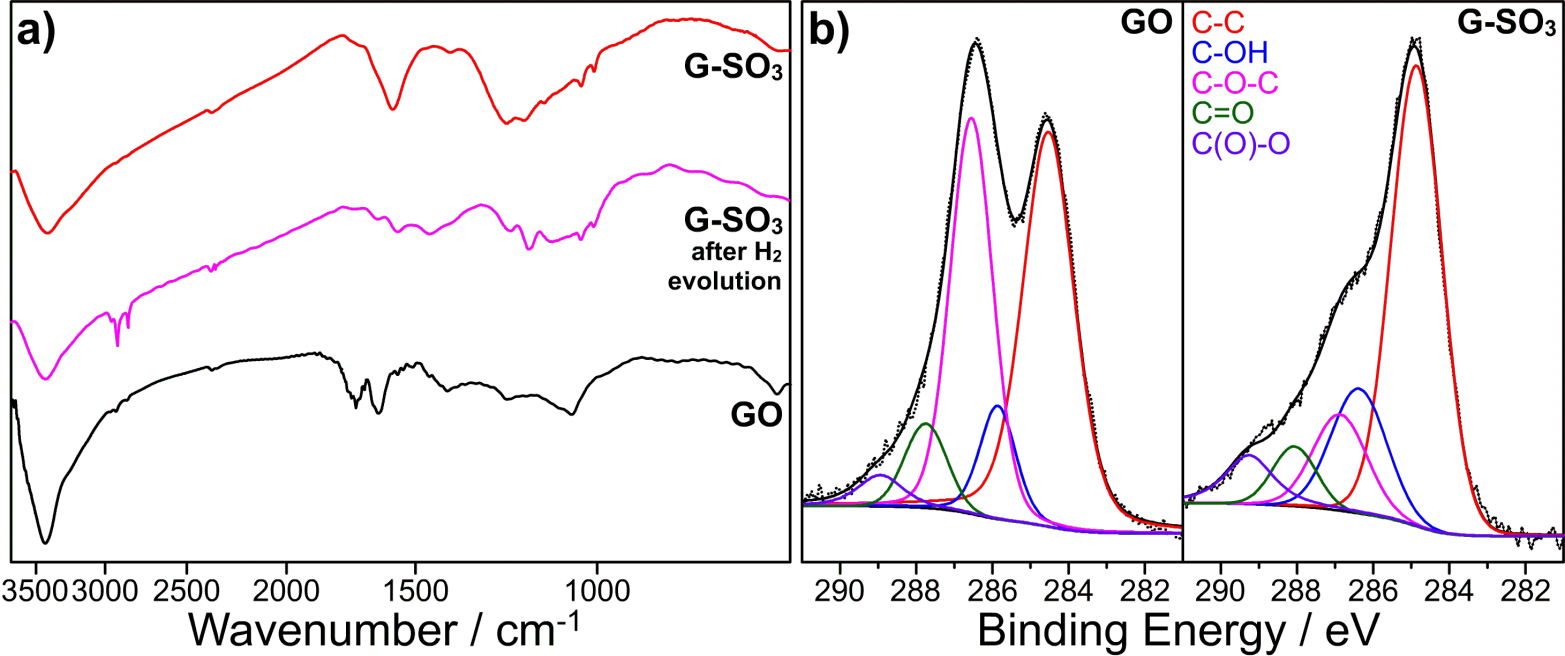
FTIR (a) and XPS (b) spectra of GO, G-SO_3_ and G-SO_3_ after photocatalytic hydrogen evolution

Fourier transform infrared spectroscopy (FTIR) is employed to characterize GO and G-SO_3_. As shown in Figure 1, compared to GO, G-SO_3_ has typical absorptions at 1177, 1123 and 1037 cm^−1^, which are assigned to ν_S-O_ and ν_S-phenyl_ confirming the modification of sulfanilic acid on graphene sheets [53]. Meanwhile, peaks attributed to C=O in carboxylic acid and carbonyl moieties (ν_C=O_ at 1720 cm^−1^), C–OH (ν_C–OH_ at 1365 cm^−1^) decrease sharply, implying a partial reduction of GO [54].

X-ray photoelectron spectroscopy (XPS) measurements were performed to confirm the differences between GO and G-SO_3_. Five different peaks centered at 284.5, 285.9, 286.6, 287.7 and 288.9 eV appear in the C1s deconvolution spectrum of GO, corresponding to C=C/C–C in aromatic rings, C–OH (hydroxy), C–O–C (epoxy), C=O (carbonyl), and C(O)O (carboxyl) groups, respectively [55]. For G-SO_3_, the peak centered at 284.6 eV becomes narrower, suggesting the partial restoration of the π-electron network in G-SO_3_. Other oxygen-containing carbon peaks, decreased sharply, indicating GO is reduced efficiently.

**Figure 2:**
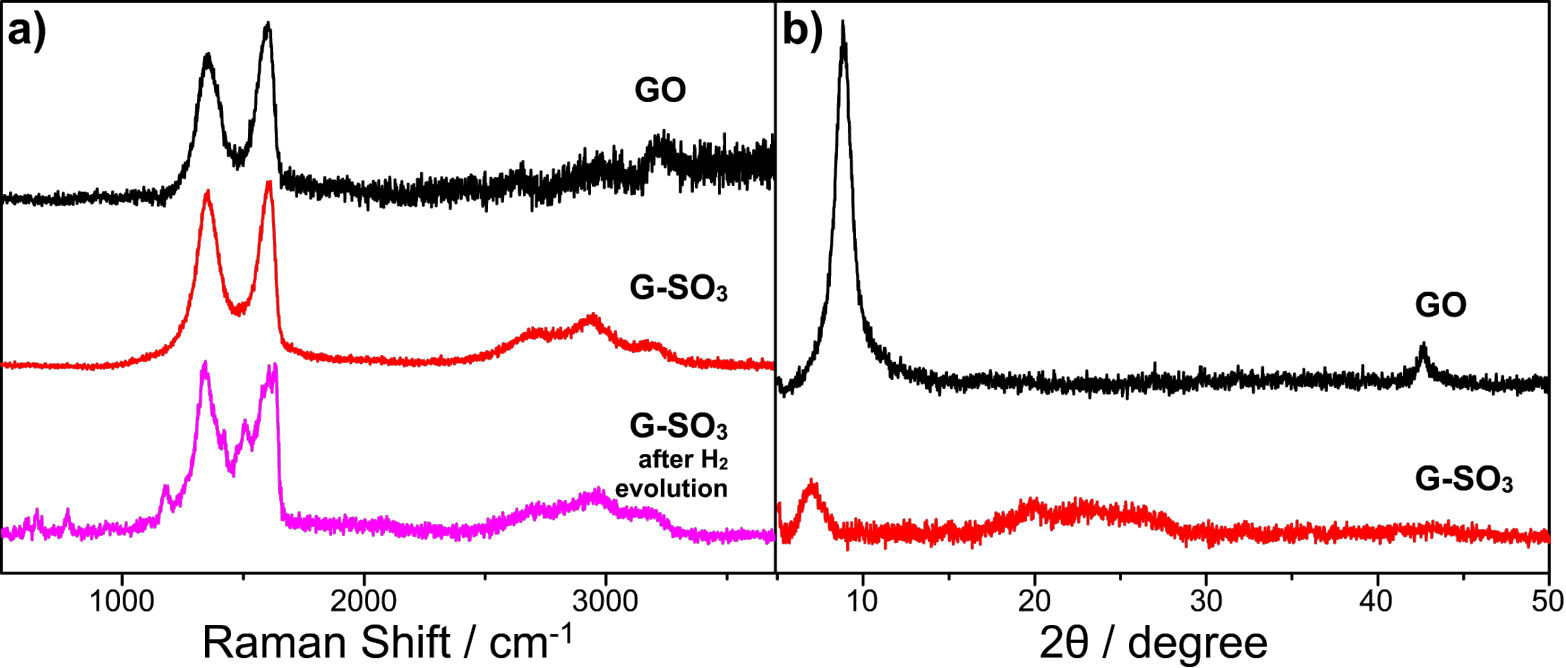
Raman (a) and XRD (b) spectra of GO (black), G-SO_3_ (red) and G-SO_3_ after photocatalytic hydrogen evolution (purple).

Further, Raman spectra and X-ray diffraction (XRD) patterns of GO and G-SO_3_ are compared in Figure 2. GO and G-SO_3_ both show the characteristic D band and G band at 1350 cm^−1^ and 1597 cm^−1^, but the enhanced *I*_D_/*I*_G_ ratio for G-SO_3_ indicates the functionalization and reduction of GO. As confirmed by the XRD patterns, after reduction and functionalization, the *d*-spacing becomes wider since the angle 2θ shifted to the left from 8.85° to 6.92°. The decreased intensity, meanwhile, manifests a more disordered structure in G-SO_3_. As a result, the obtained G-SO_3_ is both reduced and functionalized, which guarantees not only its high conductivity for electron transfer, but also its great dispersibility to act as a platform to anchor catalysts.

**Figure 3:**
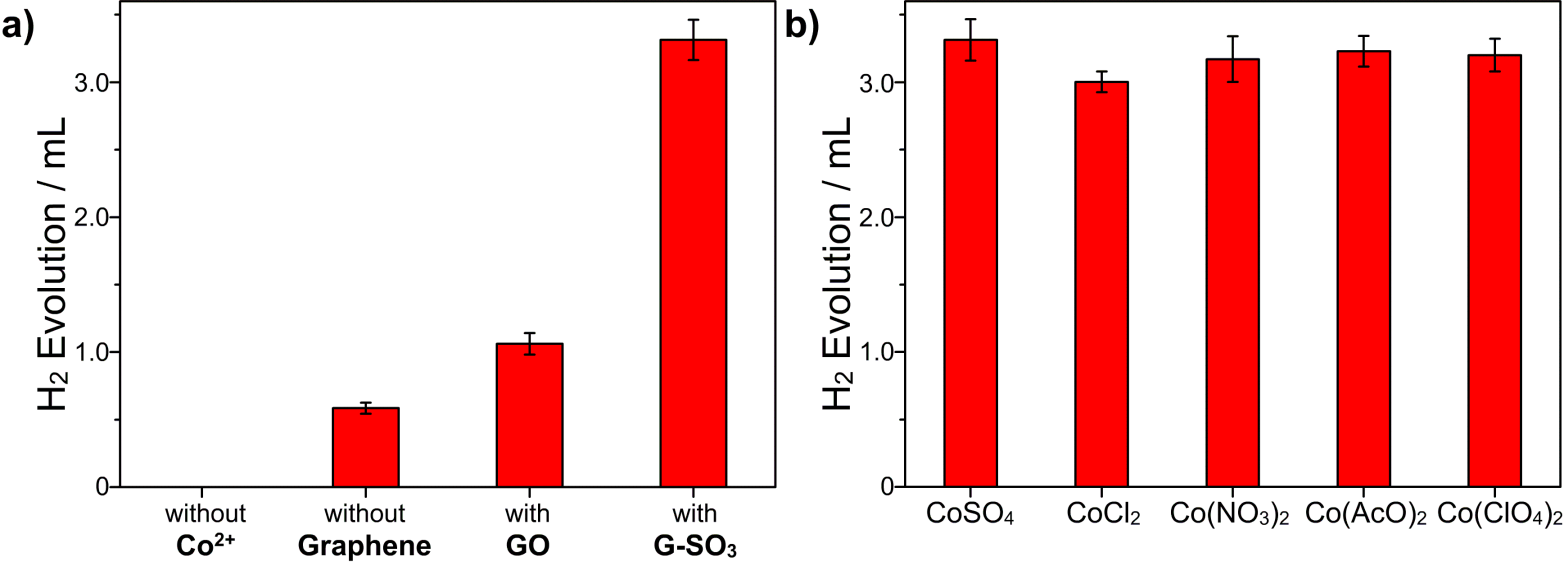
Photocatalytic hydrogen evolution with different graphene (a) and cobalt salts (b) at pH 10.86 in H_2_O after 4 h; sample concentration: Co^2+^ (2.0 × 10^−4^ mol/L), graphene (0.04 mg/mL), EY (4.0 × 10^−4^ mol/L) and TEOA (0.2 mol/L).

The photocatalytic hydrogen evolution was evaluated under irradiation at 525 nm by using TEOA as a sacrificial donor and EY as a photosensitizer, while cobalt salts and G-SO_3_ were added to serve as a catalyst in the reaction system (Figure 3). It is proposed that Co^2+^ forms a Co(TEOA)_2_^2+^ complex in the presence of TEOA [56]. No significant amounts of hydrogen were detected in the absence of either irradiation or the photosensitizer EY, indicating that hydrogen was produced through the photochemical reaction. Evidently, Co(TEOA)_2_^2+^ complexes can function as catalysts to reduce protons to hydrogen, similar to the observations of Sun and coworkers [57]. When G-SO_3_ was introduced, the amount of hydrogen obviously increased. Because our previous work [51] has demonstrated that G-SO_3_ acts as an electron mediator of EY and platinum nanoparticles co-catalyst, we consider that in the current study the electron transfer process from the EY radical anion (EY^•−^) to G-SO_3_ or in situ formed-Co(TEOA)_2_^2+^ would be facilitated. Similar to the storage phenomenon observed in carbon nanotubes, a small fraction of the electrons may get stored in graphene sheets, thus making graphene an electron reservoir to continuously provide electrons to the catalytic center [58–60]. The positive synergetic effect consequently enhances the photocatalytic activity for hydrogen evolution of the system. To examine any counter anion effects, we further used four different kinds of cobalt salts in our photocatalytic hydrogen evolution system: cobalt chloride, cobalt nitrate, cobalt perchlorate and cobalt acetate. The amounts of evolved hydrogen in each system did not differ much, indicating that the catalytic behavior is independent of the anions used. The results also manifest the formation of Co(TEOA)_2_^2+^ catalysts in the systems.

The pH value of the solution greatly influences the hydrogen evolution process of the system. The system performed well over a wide range (pH 8–12), reaching a maximal turnover at pH 10.86 (Supporting Information File 1, Figure S1). However, when pH value was below 7.2, there was no detectable hydrogen produced from the system. This pH-dependency is due to a number of factors: in acidic medium, the protonation of TEOA inevitably results in a poor electron-donating ability and less Co(TEOA)_2_^2+^ catalyst is formed. In a basic solution, the graphene dispersion was more stable and the light absorption of EY is stronger but the concentration of protons is too low.

**Figure 4:**
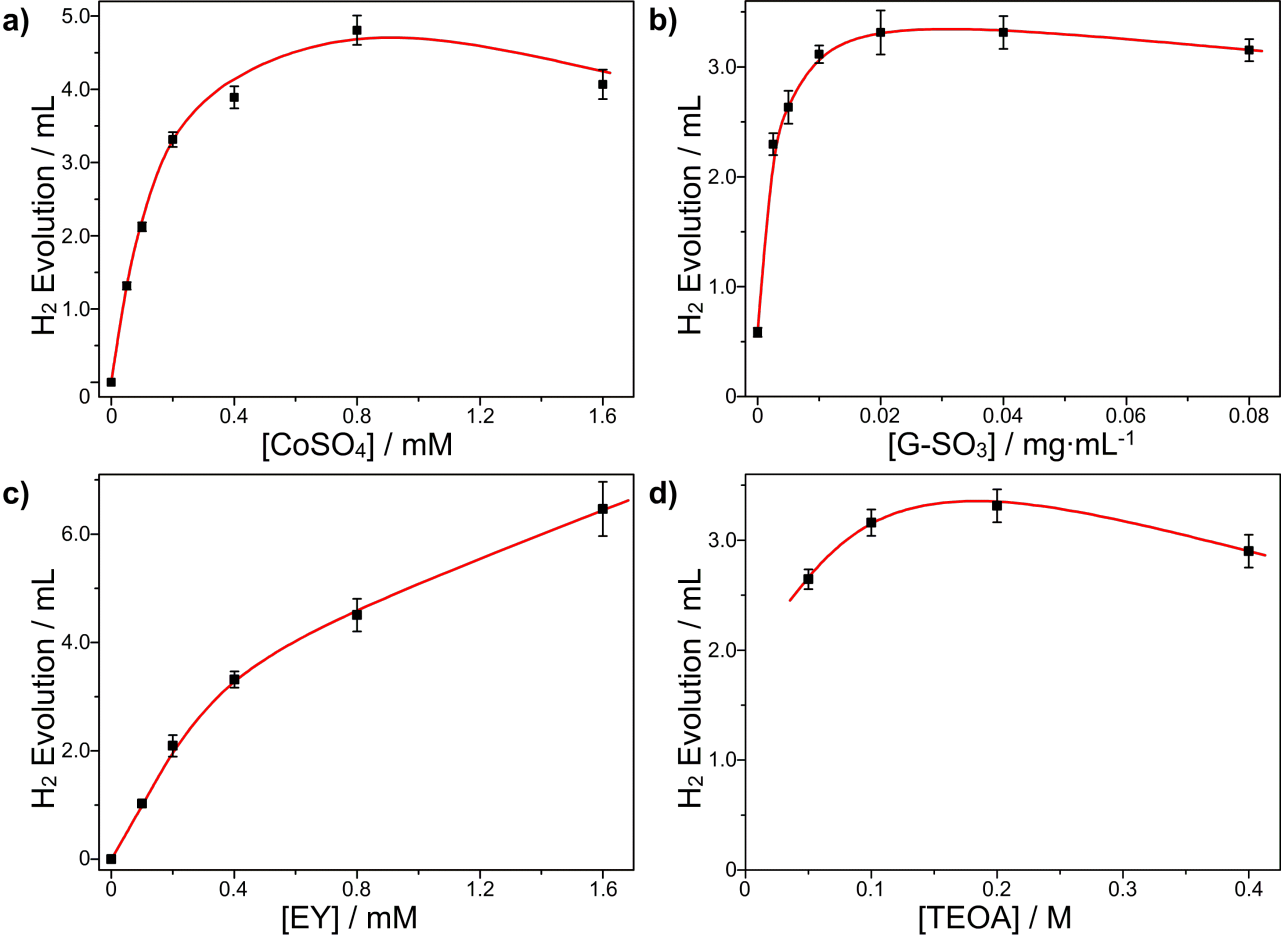
Photocatalytic hydrogen evolution as a function of the CoSO_4_ (a), G-SO_3_ (b), EY (c) and TEOA (d) concentration at pH 10.86 in H_2_O after 4 h; other sample concentration: CoSO_4_ (2.0 × 10^−4^ mol/L), G-SO_3_ (0.04 mg/mL), EY (4.0 × 10^−4^ mol/L) and TEOA (0.2 mol/L).

To optimize the hydrogen evolution system, four sets of experiments were carried out: varying the concentration of CoSO_4_, G-SO_3_, EY and TEOA used while keeping a constant concentration of the other three components at pH 10.86. The results of these experiments are shown in Figure 4. With the addition of G-SO_3_, even at low concentrations, the amount of hydrogen evolution showed a remarkable increase and reached a maximum of 3.31 mL, which is 5.6 times larger than that of the system without G-SO_3_. Further increasing the concentration of G-SO_3_ resulted in a decrease in the amount of hydrogen generated. This phenomenon happened in many other reported works, which can be explained by the light shielding effect of graphene [61–63]. Varying the concentration of CoSO_4_, a similar tendency was observed. The concentration of EY also exercises a great influence on catalytic performance of the system. The amount of hydrogen evolution increases with the concentration of EY linearly when the concentration of EY is below 0.4 mM. After a further increase of the EY concentration to 0.8 mM or 1.6 mM, however, the amount of hydrogen still increases but at a relatively slower rate. This is because self-quenching and shield-effects inevitably decrease the ability of EY to act as the photosensitizer [64]. As for the electron donor TEOA, the highest hydrogen evolution efficiency was obtained at a concentration of 0.2 M. Figure S2 in Supporting Information File 1 shows the kinetic curve of the photocatalytic hydrogen evolution under the optimized conditions at pH 10.86 (the concentration of CoSO_4_, G-SO_3_, EY and TEOA are 2.0 × 10^−4^ mol/L, 0.04 mg/mL, 4.0 × 10^−4^ mol/L and 0.2 mol/L, respectively). The total amount of hydrogen evolved under LED irradiation at 525 nm was about 3.31 mL (148 μmol) and the TON reached 148 with respect to the initial concentration of cobalt. More hydrogen was produced from the system after prolonged irradiation times but at a slower rate. The reason for the decreased rate at longer irradiation times is attributed to the decomposition of EY. As described in our previous work [51], EY decomposes to fluorescein, which has a lower absorption but a higher stability. To confirm the result in the current study, we carried out control experiments that used fluorescein as photosensitizer for hydrogen evolution under the identical condition. As shown in Supporting Information File 1, Figure S2, the rate of hydrogen evolution is the same as that of the EY system after 1 h of irradiation.

**Figure 5:**
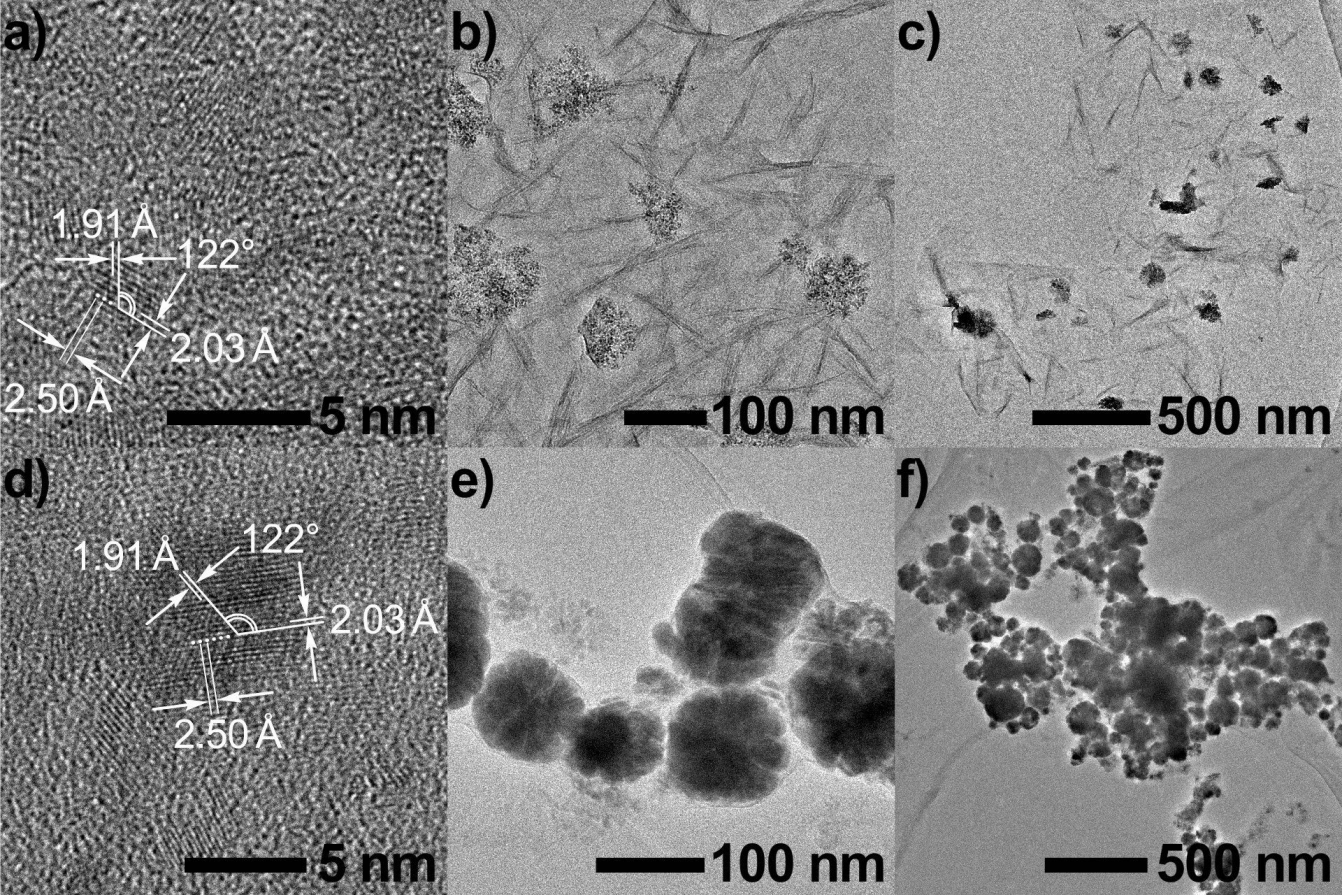
The TEM images nanoparticles after irradiation with (a–c) or without (d–f) G-SO_3_.

It is worth noting that after irradiation, a black magnetic precipitate was observed and adsorbed on the magnetron in both cases with or without G-SO_3_. When rinsed with acetone more than three times, the precipitation was visualized by TEM (transmission electron microscopy). As shown in Figure 5, in the absence of G-SO_3_ nanoparticles aggregated in size of about hundreds nanometers. Each particle is composed of lots of small nanoparticles of several nanometers in diameter. The lattice fringes in the HRTEM (high resolution TEM) images suggest a well-defined crystal structure. The lattice spacing of about 0.191 and 0.203 nm can be assigned to the (101) and (002) planes of metallic cobalt Co, space group *P*_63_/*mmc* (JCPDS card 05-0727). When G-SO_3_ was added, the TEM images exhibited much difference. Firstly, nanoparticles were formed but dispersed on G-SO_3_ sheets instead. Secondly, the sizes of the nanoparticles were smaller. The HRTEM image also showed the lattice fringes, and the lattice spacing (0.191 and 0.203 nm) is consistent with those observed in the system without G-SO_3_. This phenomenon indicated that G-SO_3_ provides a platform to support cobalt catalysts, and at the same time G-SO_3_ avoids the aggregation of the catalyst to some extent. These results are consistent with the better performance and the higher hydrogen evolution from the system with G-SO_3_.

As mentioned above, the TEM results showed that cobalt metal nanoparticles may form during the process in both cases. XPS and ICP-MS (inductively coupled plasma mass spectrometry) were used to further investigate the magnetic precipitates obtained after the hydrogen evolution reaction. XPS spectra of the precipitates with or without G-SO_3_ showed the same peak pattern and location in the range from 776 to 810 eV, corresponding to the Co 2p orbital (Supporting Information File 1, Figure S3). The cobalt lines in the spectra, however, were assigned to cobalt(II) [65], not to cobalt(0). This is different from the TEM results and in contrast to the grown cobalt metal nanoparticles on graphene [66]. ICP-MS measurements were carried out by using the precipitates obtained from the system, which gave a cobalt content of 11.1% (with graphene) and 45.0% (without graphene), respectively. Either of these results was much higher than that calculated from XPS (3.8% and 20.8%). In consideration of the fact that XPS probes only a few nanometers below the surface, the discrepancy was tentatively interpreted to be because of Co^2+^ complexes around the cobalt metal particles, which hinder the effective detection of Co metal in XPS but allows its measurement with ICP-MS. In addition, FTIR spectra of G-SO_3_ (Figure 1a, purple line) showed a typical C–H stretching vibration at 2918 cm^−1^ after photocatalytic hydrogen evolution, which apparently comes from the catalytic Co^II^(TEOA)_2_ species on the surface of G-SO_3_.

**Figure 6:**
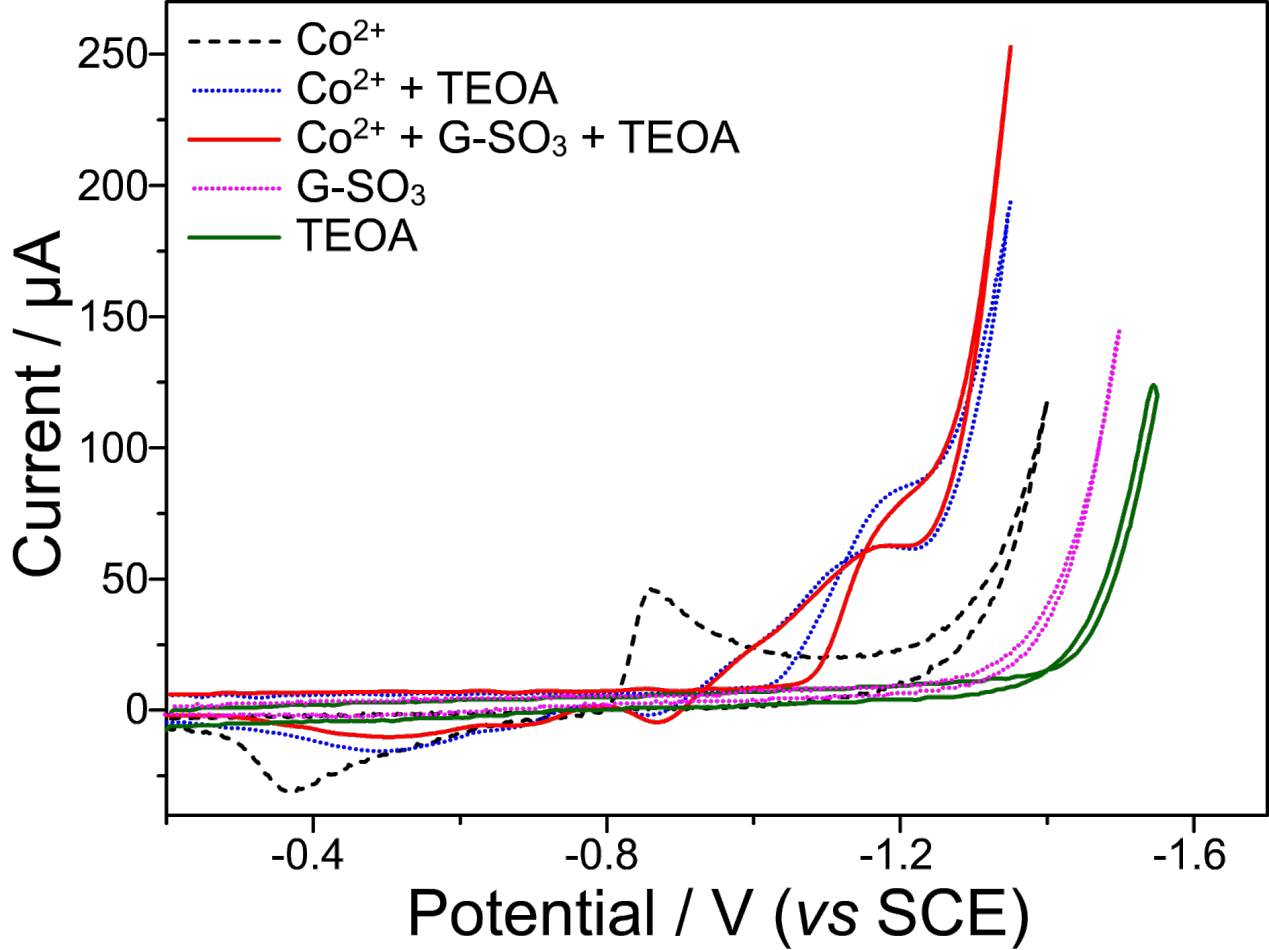
CV spectra of the 4.0 × 10^−3^ mol/L CoSO_4_ + 0.2 mol/L K_2_SO_4_ solution (black), 4.0 × 10^−3^ mol/L CoSO_4_ + 0.4 mol/L TEOA + 0.2 mol/L K_2_SO_4_ solution (blue), 4.0 × 10^−3^ mol/L CoSO_4_ + 0.4 mol/L TEOA + 0.04 mg/mL G-SO_3_ solution + 0.2 mol/L K_2_SO_4_ (red), 0.04 mg/mL G-SO_3_ solution + 0.2 mol/L K_2_SO_4_ (purple) and 0.4 mol/L TEOA + 0.2 mol/L K_2_SO_4_ solution (green).

Cyclic voltammetry (CV) spectra were used to investigate the hydrogen evolution system (Figure 6). And the results showed that Co(TEOA)_2_^2+^ complex was active for electrocatalytic hydrogen evolution in 0.2 M K_2_SO_4_ and 0.4 M TEOA aqueous solution. The Co^II^(TEOA)_2_/Co^I^(TEOA)_2_ reduction band peaked at about −1.1 V (vs SCE), and is followed by a rapid rise in current at −1.25 V (vs SCE). This increase of current, accompanied by the evolution of bubbles, can be attributed to the catalytic generation of hydrogen from the aqueous solution [67]. In order to verify that Co(TEOA)_2_^2+^ is responsible for the catalysis, control experiments were performed at room temperature. When the 0.2 M K_2_SO_4_ aqueous solution or 0.2 M K_2_SO_4_ and 0.4 M TEOA aqueous solution were studied, no catalytic current appeared until the potential was over −1.5 V (vs SCE). When G-SO_3_ was added, no new peak emerged, but the catalytic current intensity increased by about 20%. The observation implied that in the presence of G-SO_3_, electron transfer processes become faster, which results in a higher activity toward electrocatalytic hydrogen evolution. Analogously, G-SO_3_ is important for enhancing the performance of photocatalytic hydrogen evolution. For photocatalytic hydrogen evolution systems, the photosensitizer EY is often reduced by TEOA to form EY^•−^ radical anions. Since the oxidation potential of EY^•−^ (−1.05 V vs NHE) [68] is more negative than that of Co^II^(TEOA)_2_/Co^I^(TEOA)_2_ couple, an electron transfer from EY^•−^ to cobalt-center is thermodynamically feasible and initiates the whole hydrogen evolution process.

**Scheme 1:**
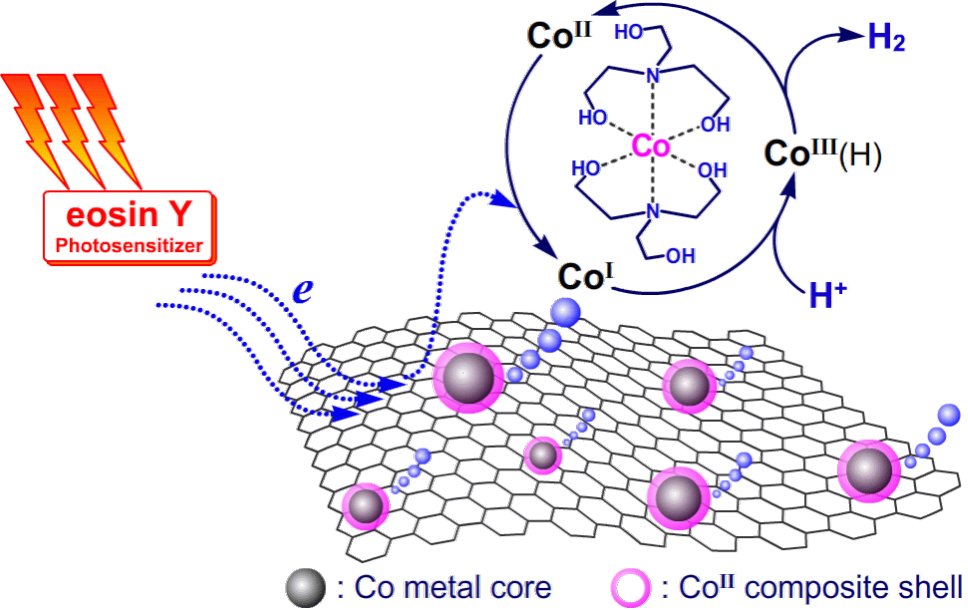
Schematic illustration of the photocatalytic hydrogen evolution process.

Taking into consideration all results of the TEM, XPS, ICP–MS and CV measurements, the photocatalytic process in this work can be described in Scheme 1. When all the components (TEOA, EY, G-SO_3_, CoSO_4_) were added into the reaction system, Co^II^(TEOA)_2_ complexes were formed in situ and are at well-adsorbed or surround the G-SO_3_. In fact, not all of the Co^II^(TEOA)_2_ complexes were on the surface of G-SO_3_, because ICP-MS measurements gave a cobalt content of 11.1%, which was much lower than the feeding ratio of 22.8%. Upon irradiation, the electrons of the EY^•−^ radical anion generated from EY and TEOA, transfer to G-SO_3_ or directly to Co^II^(TEOA)_2_ to initiate the catalytic hydrogen evolution. Since graphene is an ideal electron acceptor and/or electron reservoir, an efficient multi-electron transfer toward the catalytic center Co^II^(TEOA)_2_ takes place. Regarding the reports about photocatalytic hydrogen evolution systems based on molecular cobalt complexes in the literature [20], it could be speculated that in the present work the reduction of Co^II^(TEOA)_2_ to Co^I^(TEOA)_2_ occurs firstly. Co^I^(TEOA)_2_, on the one hand, can be protonated to form Co^III^(TEOA)_2_H hydride, which reacts with another hydride to eliminate hydrogen or further protonated to release hydrogen and Co^III^(TEOA)_2_, which is subsequently reduced to Co^II^(TEOA)_2_ for the next catalytic circulation. On the other hand, the protonated Co^III^(TEOA)_2_H can also be reduced further to yield Co^II^(TEOA)_2_H hydride, which experienced the above cycle for hydrogen evolution. Specifically, if the Co^I^(TEOA)_2_ species is not protonated at low concentrations of protons in the system, it can be reduced further to Co^0^(TEOA)_2_ [69]. Since there are ligands around Co^0^(TEOA)_2_, this Co^0^(TEOA)_2_ species can be protonated to form Co^II^(TEOA)_2_H that would either eliminate hydrogen as discussed above or release ligands to form metallic cobalt. The obtained metallic cobalt may function as nucleation center anchoring other cobalt-catalysts.

## Conclusion

In summary, we introduce a new water-soluble graphene-cobalt-based hydrogen evolution system. With TEOA as the electron donor, EY as the photosensitizer, Co(TEOA)_2_^2+^ formed in situ from cobalt salts and TEOA on the surface of G-SO_3_ or around it as the initial catalyst, the effective hydrogen evolution system is established. By using 525 nm LEDs as the light source, this system shows a 5.6 times higher efficiency than that of the same system without G-SO_3_, and the hydrogen can continually evolve even after 20 h. With TEM, ICP-MS, and XPS measurements the magnetic precipitation after irradiation is confirmed to be Co metal surrounded by Co^2+^ species. CV results indicate the redox potential for the Co^II^(TEOA)_2_/Co^I^(TEOA)_2_, manifesting the feasible electron transfer process thermodynamically. The effects of the pH value, as well as the concentration of G-SO_3_, CoSO_4_ and TEOA were investigated in detail not only to optimize the catalytic activity for hydrogen evolution but also to understand the reaction mechanism. The enhanced activity of the photocatalytic system makes it attractive to design and synthesize new catalysts by using graphene and earth-abundant metal salts for the photocatalytic H_2_ production.

## Supporting Information

File 1 Experimental part.
