# Supplementary material for: Enhanced photocatalytic hydrogen evolution by combining water soluble graphene with cobalt salts
Source: Beilstein J Nanotechnol. 2014 Jul 29;5:1167–74. doi: 10.3762/bjnano.5.128 (PMC4142871; doi:10.3762/bjnano.5.128)
Supplement: File 1 — Experimental part. [file Beilstein_J_Nanotechnol-05-1167-s001.pdf]

Supporting Information

for

Enhanced photocatalytic hydrogen evolution by combining water soluble graphene with cobalt salts

Jing Wang, Ke Feng*, Hui-Hui Zhang, Bin Chen, Zhi-Jun Li, Qing-Yuan Meng, Li-Ping Zhang, Chen-Ho Tung and Li-Zhu Wu*

Address: Key Laboratory of Photochemical Conversion and Optoelectronic Materials, Technical Institute of Physics and Chemistry & University of Chinese Academy of Sciences, the Chinese Academy of Sciences, Beijing 100190, P. R. China

Email: Li-Zhu Wu* - lzwu@mail.ipc.ac.cn; Ke Feng* - kefeng@mail.ipc.ac.cn

* Corresponding author

Experimental

Instruments

The TEM images were taken on a JEOL JEM 2100F transmission electron microscope with an accelerating voltage of 200 kV. The Raman spectrum was recorded on a Via-Reflex Raman system using a 532 nm excitation wavelength. The XPS measurements were performed on an ESCALAB 250 spectrophotometer with Al-K*α* radiation. FTIR spectra were taken on Excalibur 3100 system (Varian, USA). Powder XRD was performed on a Bruker D8-Advance X-ray diffractometer with monochromatized Cu Kα radiation (λ = 1.5418 Å). The composition of material was determined by inductively coupled plasma mass spectroscopy (ICP-MS, Varian 710-ES, USA). A three-electrode system, a 3 mm glass carbon working electrode, a platinum wire counter electrode, and a SCE reference electrode, was used to measure the cyclic voltammograms with 0.2 M K_2SO_4 as electrolyte.

Experimental details

Synthesis of GO and water-dispersible sulfonated-graphene G-SO_3

GO is synthesized by the modified Hummers' method from graphite. The graphite powder was pretreated with $K_2S_2O_8$/P_2O_5/concentrated H_2SO_4, and then oxidized by H_2SO_4/$KMnO_4$ consistent with our previous work [1].

G-SO_3 is obtained as follows. In brief, 40 mL 0.5 mg/mL GO suspension was first reduced with 160 mg $NaBH_4$ (4.22 mmol) at 80 °C for 1 h. 0.7 g (4 mmol) sulfanilic acid was dissolved in 50 mL water in a 100 mL round-bottom flask, then 9.2 mL 1M

HCl was added. 0.3 g $NaNO_2$ (4.35 mmol) dissolved in 5 mL water was dropped slowly to the above flask in ice bath. After reacting for 1 h, the obtained RGO suspension was added to the flask, and the mixture was maintained at 0 °C for 4 h, and then kept stirring overnight at room temperature.

Photocatalytic hydrogen evolution measurements

A typical procedure for hydrogen production is as follows. Aqueous suspension of G-SO_3, $CoSO_4$, EY (Eosin Y) and TEOA (triethanolamine) were added to a Pyrex tube. The amount of each component is according to the determined condition experiments. The total volume is adjusted to 10 mL. The pH value of the mixed solution was determined by a pH meter and was adjusted by the addition of aqueous NaOH or HCl solution. The sample was irradiated under 525 nm LEDs. The generated photoproduct of H_2 was characterized by GC analysis (Shimadzu 14B).

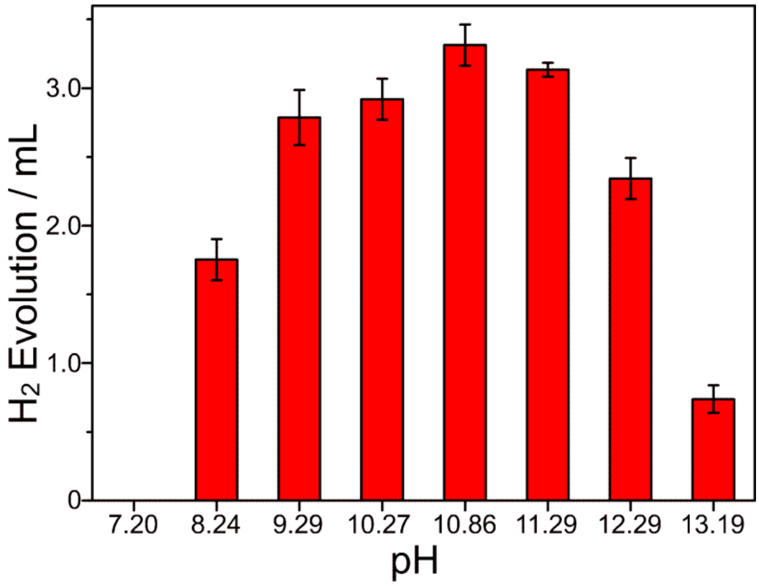

Figure S1: Photocatalytic hydrogen evolution at different pH in H_2O in 4 h; sample concentration: $CoSO_4$ (2.0 × 10^{-4} mol/L), G-SO_3 (0.04 mg/mL), EY (4.0 × 10^{-4} mol/L) and TEOA (0.2 mol/L).

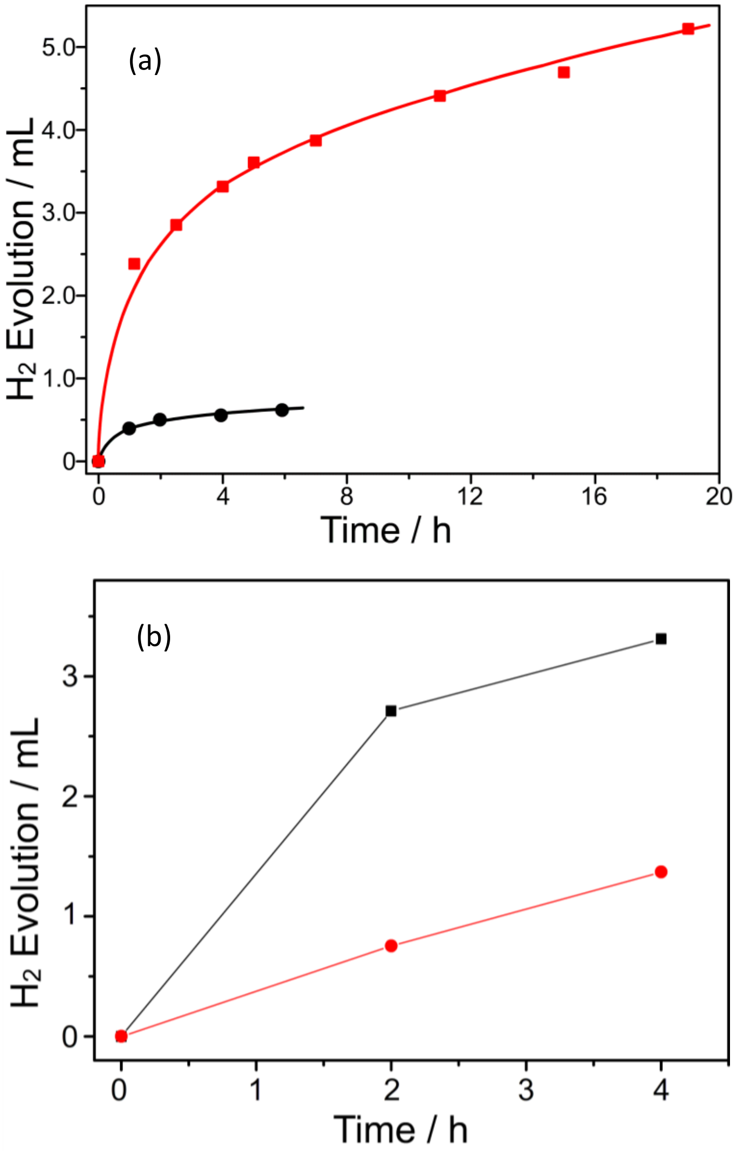

Figure S2: (a) Kinetic curves of the photocatalytic hydrogen evolution with the optimized condition (the concentration of $CoSO_4$, G-SO_3, EY and TEOA are 2.0×10^{-4} mol/L, 0.04 mg/mL, 4.0×10^{-4} mol/L and 0.2 mol/L, respectively, the pH value 10.86) with (red) and without (black) G-SO_3. (b) Photocatalytic hydrogen evolution with different photosensitizer under the optimal condition (the concentration of $CoSO_4$, G-SO_3, EY (or fluorescein), and TEOA are 2.0×10^{-4} mol/L, 0.04 mg/mL, 4.0×10^{-4} mol/L and 0.2 mol/L, respectively, at pH 10.86): EY (black) and fluorescein (red) as the photosensitizer, respectively.

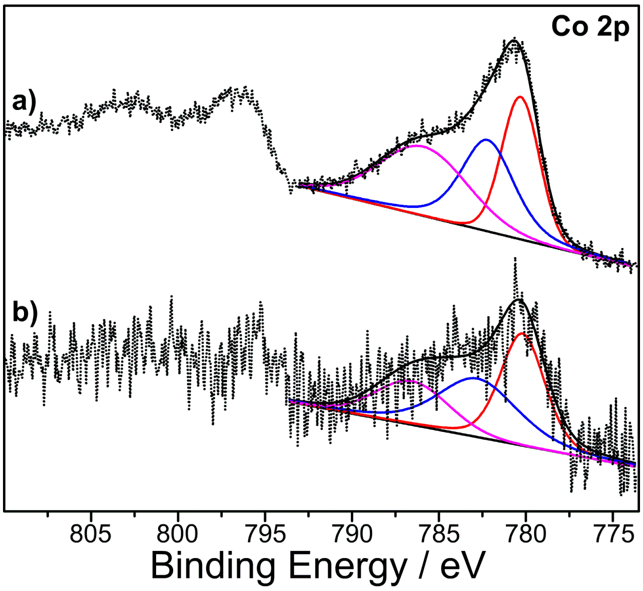

Figure S3: XPS spectra of Co 2p after irradiation: (a) without and (b) with G-SO_3.

References

1. Zhang, H.-H.; Liu, Q.; Feng, K.; Chen, B.; Tung, C.-H.; Wu, L.-Z. *Langmuir* **2012,** *28,* 8224–8229. doi:10.1021/la301429g
